# Oxytocin Release Increases With Age and Is Associated With Life Satisfaction and Prosocial Behaviors

**DOI:** 10.3389/fnbeh.2022.846234

**Published:** 2022-04-21

**Authors:** Paul J. Zak, Ben Curry, Tyler Owen, Jorge A. Barraza

**Affiliations:** ^1^Center for Neuroeconomics Studies, Claremont Graduate University, Claremont, CA, United States; ^2^TripActions, San Francisco, CA, United States; ^3^Department of Psychology, University of Southern California, Los Angeles, CA, United States

**Keywords:** positive psychology, lifespan, neuroendocrinology, behavior, gratitude

## Abstract

Helping behaviors and life satisfaction generally increase after middle-age. Identifying the neural substrates of prosocial behaviors in older adults may offer additional insights into these changes over the lifespan. The present study examines the endogenous release of the neuromodulator oxytocin (OT) in participants aged 18–99 and its relationship to prosocial behaviors. OT has been shown to influence trust, altruism, charity, and generosity, yet the effect of age on OT release has not been well-established. Blood samples before and after a video stimulus were obtained from 103 participants in order to examine the impact of OT on prosocial behaviors. We found that OT release following a social prime increased with age (*r* = 0.49, *p* = 0.001) and that OT moderated the relationship between age and donations to charity. We tested for robustness by examining three additional prosocial behaviors, money and goods donated to charity during the past year and social-sector volunteering. OT moderated the impact of age on all three prosocial behaviors (*ps* < 0.05). The analysis also showed that participants’ change in OT was positively associated with satisfaction with life (*p* = 0.04), empathic concern (*p* = 0.015), dispositional gratitude (*p* = 0.019), and religious commitment (*p* = 0.001). Our findings indicate that the neural chemistry that helps sustain social relationships and live a fulfilled life appear to strengthen with age.

## Introduction

The physical effects of aging on the body have been well documented (Saxon et al., 2014). Similarly, the effect of aging on cognitive function has been extensively studied (Meaney et al., 1995; Conrad and Bimonte-Nelson, 2010). For example, aging impairs efficiency and processing speed (Salthouse, 1994, 1996; Li et al., 2004), explicit learning (Salthouse, 2006), working memory (Salthouse, 1992; McArdle et al., 2004), and attention and problem-solving (Craik and Salthouse, 2000). These changes may result from the reduction of gray matter volume in brain regions as one ages (Brown and Ridderinkhof, 2009), including the anterior cingulate cortex, the orbital frontal cortex, the superior temporal sulcus, the insula, and the dorsal and ventral striatum (Matochik et al., 2000; Raz et al., 2000; Good et al., 2001; Sowell et al., 2003; Allen et al., 2005; Walhovd et al., 2005).

Social behaviors and emotional responses also change with age (Midlarsky et al., 2015; Matsumoto et al., 2016; Pornpattananangkul et al., 2019). Seniors spend more time volunteering and donate a larger proportion of their income to charity compared to younger adults (Simmons and Emanuele, 2004; Omoto et al., 2009; Choi and Chou, 2010; Bekkers and Wiepking, 2011; Wiles and Jayasinha, 2013). Both of these effects are stronger for religiously active older adults (Lee and Chang, 2007; Bekkers and Wiepking, 2011). In laboratory studies, seniors share more money with strangers than younger adults (Beadle et al., 2015) with and without empathy priming (Matsumoto et al., 2016; Rosen et al., 2016; Rosi et al., 2019).

A number of factors are associated with increased prosociality in seniors compared to younger cohorts. These include increased positive affect (Stanley and Isaacowitz, 2011), the use of intuition rather than calculation during decisions (Rosi et al., 2019), and improved emotional regulation (Larcom and Isaacowitz, 2009; Charles and Carstensen, 2010; Samanez-Larkin and Carstensen, 2011). Older adults may develop skills that improve their moods that younger people are less likely to use such as gazing at happy faces (Isaacowitz et al., 2009) though there is significant variation across individuals (Stanley and Isaacowitz, 2012). Age-related positivity also manifests in older adults as they attend to positive information more than negative information (Reed and Carstensen, 2012). Older adults are also better at reasoning about interpersonal and intergroup conflicts (Artistico et al., 2003; Thornton and Dumke, 2005; Grossmann et al., 2010), and have more accurate evaluations of their own knowledge (Kovalchik et al., 2005). Prosocial behaviors in seniors, relative to younger people, may be more likely to arise because of heightened state-induced affective empathy (Ze et al., 2014; Rosen et al., 2016; Sun et al., 2018; Bailey et al., 2021). Yet, no differences are found in trait empathy between older and younger adults (Konrath et al., 2011; O’Brien et al., 2013; Sun et al., 2018).

Age-varying neurochemical responses might help explain seniors’ prosociality. Correlational and experimental studies of prosocial behaviors suggest oxytocin (OT) as a likely candidate. The release of OT is associated with empathic concern (Barraza and Zak, 2009, 2013) while synthetic OT administration enhances empathy (Hurlemann et al., 2010; Shamay-Tsoory et al., 2013). Both the change in endogenous OT and synthetic OT administration have been associated with donations to charity (Barraza and Zak, 2009; Barraza et al., 2011), generosity toward strangers (Zak et al., 2007; Pornpattananangkul et al., 2017), and trustworthiness (Zak et al., 2004, 2005; Terris et al., 2018) among other prosocial behaviors (Barraza and Zak, 2013) though generosity after OT administration may be biased toward in-groups (Ten Velden et al., 2017) and OT administration may or may not affect trust (Kosfeld et al., 2005; Declerck et al., 2020). In addition, the effect of OT infusion on recognizing emotions in faces shows a positive age gradient (Campbell et al., 2014; Ebner et al., 2015a,b; Horta et al., 2019).

Evidence for the effect of OT on social-emotional responses in older adults comes primarily from intranasal OT administration studies, albeit with mixed findings (Horta et al., 2020). For instance, single-dose OT administration improves emotional recognition in healthy older men (Campbell et al., 2014) and in dementia patients (Jesso et al., 2011). However, replication using a larger sample and additional emotional recognition tasks failed to replicate this finding (Grainger et al., 2018) and two meta analyses show variable effects (Shahrestani et al., 2013; Leppanen et al., 2017). OT administration improves self-reported mood (Ebner et al., 2015a,b) and enhances resting state amygdala-mPFC coupling (Ebner et al., 2013) in older men but not older women. A three-dose OT administration study in a sample of older adults with dementia demonstrated reduced apathy, increased expressions of empathy, and improved caregiver interactions (Finger et al., 2015). A longer (10-day) trial of intranasal OT administration in healthy older adults (mean 80 years) revealed improved dispositional gratitude and reduced fatigue (Barraza et al., 2013).

While synthetic OT administration in older adults may have clinical value, such an intervention may be premature until endogenous OT release in seniors is understood. Some reports find basal OT declines with age with a larger effect for women compared to men (Plasencia et al., 2019), although the opposite effect has also been reported (Kunitake et al., 2020). The current study measures OT reactivity in response to a video stimulus with social content because it is the release of OT that has been associated with prosocial behaviors and the quality of relationships (Zak, 2012). Basal OT is generally unpredictive of behavior in healthy adults (Cochran et al., 2013). The reactivity of peripheral OT to social stimuli occurs within 1 s of stimulus onset and reflects a change in central OT via hypothalamic control (Valstad et al., 2017). The correspondence between the change in central and peripheral OT is the reason why a change in OT measured in blood is related to behaviors (Zak et al., 2004, 2005; Zak, 2012; Barraza and Zak, 2013; Terris et al., 2018). Of interest for the present study, OT-expressing neurons appear to be unaffected by age (Wierda et al., 1991; Ishunina et al., 1999) suggesting that OT is a feasible candidate to influence the age gradient in prosociality.

If OT release in seniors is greater than that in younger adults and is associated with prosociality, it may also be related to the age gradient in satisfaction with life (SWL). Many, though not all, studies report that SWL rises after middle-age and stays high until an advanced age (Diener and Suh, 1997; Diener, 2009; Hansen and Slagsvold, 2012; Jivraj et al., 2014). Part of this effect is due to improved coping skills in the face of stress (Hamarat et al., 2002). More generally, life satisfaction among seniors covaries with health, marital status, religiosity, and cognitive function (Bergan and McConatha, 2001; Bailey and Snyder, 2007; Parra-Rizo and Sanchis-Soler, 2020). Basal OT has been associated with higher SWL in young adults (Garforth et al., 2020) but has not to date been examined in older populations.

The study reported here was designed to test the hypothesis that socially primed OT release will increase with age. We also hypothesized that the increase in OT will positively correlate with changes in age-related prosocial behaviors as well as with participants’ SWL. These three hypotheses seek to shed light on the neural mechanisms that influence social behaviors of older adults compared to younger ones.

## Materials and Methods

### Participants

Participants were 103 students from the Claremont Colleges, residents from Claremont, CA and neighboring cities in Southern California, and older adults from nearby independent living communities. These participants ranged in age from 18 to 99 (*M* = 50.39, SD = 25.53; 57.3% female). Participants were moderately diverse, self-identifying as Caucasian (70.9%), Latino/Hispanic (7.8%), African American/Black (3.9%), Asian (2.9%), Pacific Islander (2.9%), and mixed ethnicity/other (11.7%). Participants were divided into young (18–35, *n* = 41), middle (36–65, *n* = 28), and older (65–99, *n* = 34) adult cohorts for comparison. Using the average size effect and standard errors for the change in OT in Barraza and Zak (2009) for 103 observations produces a power of test of 0.97 using G*power (Faul et al., 2007) indicating the sample size is sufficient to detect the hypothesized effects.

### Procedure

This study was approved by the Claremont Graduate University Institutional Review Board (IRB # 1255). Written informed consent was obtained from all participants prior to inclusion in the study. Participants were assigned an alphanumeric code to mask their identities and there was no deception of any kind. Tasks were monetarily incentivized such that participants could earn up to $40 depending on their decisions and the decisions of others. A lab administrator who was not associated with the study paid participants their earnings in private to maintain anonymity.

#### Study Timeline

**FIGURE 1:**
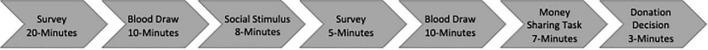
Timeline of the experiment.

Figure 1 shows how participants progressed through the study. After consent, participants completed surveys regarding their opinions, attitudes, and demographics. Then, a basal blood sample was obtained after which participants watched a short video. After watching the video, participants rated the emotions they were feeling and a second blood draw was done. Participants then made two decisions involving money to measure prosociality. The first involved sharing money with a stranger in the lab that day and the second was a decision to donate some of their earning to charity.

#### Video Stimulus

Participants watched a 100-s video of a father describing his feelings about his 2-year-old son who is dying from brain cancer. The video shows the child playing in the hospital while his father talks. The video was used with permission from St. Jude Children’s Research Hospital. The video is described in detail elsewhere (Barraza and Zak, 2009; Barraza et al., 2015) and has been shown to consistently stimulate OT release and elicit charitable donations (Barraza and Zak, 2013). Since the goal is to induce an increase in OT, no control stimulus is needed following previous studies that expose participants to experiences that may induce an increase in OT (Zak et al., 2004, 2005; Alexander et al., 2015; Curry et al., 2015; Terris et al., 2018).

#### Surveys

Participants completed surveys to assess demographics and social behaviors as controls. Another control measures is trait empathy (Interpersonal Reactivity Index, IRI; Davis, 1983) that can affect prosocial behaviors (Rameson et al., 2012). A standard 5 question assessment captured satisfaction with life (SWL; Diener et al., 1985) and in order to examine if religion affected prosocial behaviors and SWL, the 10 question religious commitment inventory was used to assess religious activity (RCI; Worthington et al., 2003). Dispositional gratitude was measured using the GQ-6 (McCullough et al., 2002) because gratitude increased in an OT infusion study (Barraza et al., 2013) and influences prosocial behaviors (Bartlett and DeSteno, 2006).

#### Prosocial Behaviors

In order to obtain an observable prosocial behavior, participants were given an opportunity to donate some of their earnings from an unrelated task (Range: $25-$40, M: $25.62, SD: $2.47) to St. Jude Children’s Research Hospital. The video presents the story of a father and his 2-year old son who has cancer. Donation are not solicited in the video. Participants were handed a paper receipt noting their earnings from an interpersonal money sharing task done after video and second blood draw (Figure 1) with a statement offering a choice to make a donation from their earnings to St. Jude. Neutral language was used when describing the donation and the decision was made in private. Participants then carried the receipt to a cashier for payment. At the end of the study, all donations were sent to St. Jude. Previous research using the St. Jude video (Barraza and Zak, 2009) showed that donations are made when participants had increases in OT as well as a marker of attention to, or interest in, the stimulus, cortisol (CORT). Subsequent research has shown that for short videos like the one used here, the faster acting arousal biomarker adrenocorticotropin hormone (ACTH) is a more effective measure (Lin et al., 2013). ACTH is the principle pituitary signal to release CORT from the adrenals and the latter provides a second measure of the arousal response due to the video stimulus. In order to ensure the neurochemical response was captured, OT, CORT, and ACTH were measured before and after the video.

Additional evidence of prosocial behaviors was measured with three questions from the intake questionnaire. Participants were asked to report for the previous year how much money they donated to charity, the value of goods donated to charity, and the number of times they had volunteered at a social sector organization. These measures used ordinal scales from 1 to 5 that were defined for participants.

#### Blood Draws and Assays

Participants received two 28 ml blood draws from an antecubital vein by a qualified phlebotomist. Two 8-ml EDTA whole-blood tubes and one 12 ml serum-separator tube were drawn in a sterile field using Vacutainer^®^ blood-collection kits. After phlebotomy, each tube was immediately stored on ice before being placed in a refrigerated centrifuge and spun at 1,500 rpm at 4°C for 12 min following previously published protocols (Zak et al., 2005). Plasma and serum were aliquoted into 2-ml polypropylene Fisher brand microtubes. The microtubes were immediately placed on dry ice and then transferred to an −80°C freezer until analysis.

All hormones were assayed using either radioimmunoassay (RIA) or enzyme-linked immunosorbent assays (ELISA). ACTH (plasma-RIA) samples were assayed using a kit produced by DiaSorin, Inc. (Stillwater, MN, United States); CORT (serum-RIA) samples were assayed using a Diagnostic Systems Laboratories (Webster, TX, United States) kit. OT was assayed using a competitive ELISA assay from Assay Designs, Inc. (Ann Arbor, MI, United States). All tests were performed by the Endocrine Core Laboratory of the Yerkes National Primate Research Center at Emory University (Atlanta, GA, United States). The inter-assay CVs < 15% for all analytes.

Oxytocin assays were not put through an extraction step. While there has been debate on the necessity of extraction (Szeto et al., 2011; McCullough et al., 2013; Leng and Sabatier, 2016), unextracted plasma OT has been shown to be reliable (Plasencia et al., 2019; Chu et al., 2020) and may have a stronger association with psychological factors than extracted OT (MacLean et al., 2019; Saxbe et al., 2019). Using an ELISA also permits comparison to an earlier study using the same video stimulus (Barraza and Zak, 2009) and studies of OT in older adults (Plasencia et al., 2019). Since the need for an extraction step in the OT assay is unclear, we sought to confirm our results by running an assay with extraction for approximately one-third of participants for whom there was sufficient additional plasma. This was done using an RIA kit (Bachem, Inc., Torrance, CA, United States). Our analyses exclusively uses the percentage change in OT in plasma to reflect the release of central OT, reducing likelihood of bias due to the type of assay.

#### Statistical Analysis

Student’s *t*-tests were used to examine the hypothesized relationship between the change in OT, age, donation decisions, and SWL. The cited literature identifies ACTH and/or CORT as part of the neural responses to video stimuli that are associated with charitable donations so these are included as covariates. In order to establish inferential validity of the observed prosocial measure, in-experiment donations to charity, data from three self-report prosocial behaviors were also measured, donations of goods, time, and money. This convergent validation, known as triangulation, reduces the likelihood of making a Type 1 error (Fielding, 2012; Loken and Gelman, 2017). The self-report data are ordinal but have enough variation to permit parametric analyses; that is, we cannot reject that with all three are Gaussian using the Jarque-Bera test [donated money: χ(2) = 3.81, *p* = 0.149; donated goods: χ(2) = 3.031, *p* = 0.220; donated time: χ(2) = 4.59, *p* = 0.101]. The moderating impact of age and OT release on prosocial behaviors was examined using least squares and ordered logit regressions. Following the literature relating religiosity and SWL, robustness was examined by examining the relationship between the change in OT and RCI and by including RCI as a covariate in analyses of prosocial behaviors.

## Results

### Physiology

**FIGURE 2:**
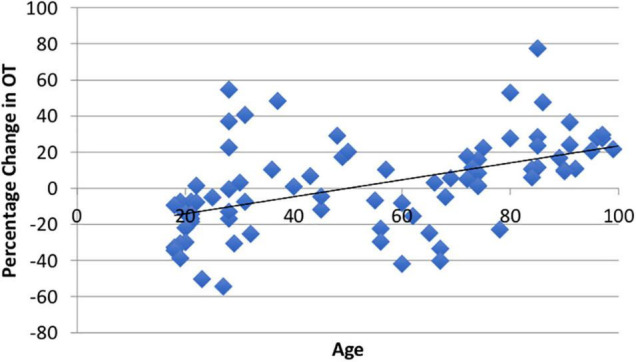
There was a significant positive relationship (*r* = 0.49, *p* = 0.001) between age and ΔOT from the video stimulus. The video stimulus increased OT in 55% of the sample.

Using paired *t*-tests, we found that watching the video produced significant within-subject changes in both ACTH (*M* = 9.05%, SD = 45.32%, *t*(94) = 1.66, *p* = 0.05) and CORT (*M* = −9.5%, SD = 34.72%, *t*(81) = 3.17, *p* = 0.001). The average ΔOT using the larger ELISA sample was heterogeneous (M1 = 371.56, SD1 = 315.72; M2 = 372.41, SD2 = 273.36) as reported in other studies (Terris et al., 2018) and was not different than zero (*M* = 1.81%, SD = 25.91, *t*(86) = 0.08, *p* = 0.53; Appendix Figure A1). The change in all three biomarkers did not show sex differences (*ps* > 0.20). Consistent with our hypothesis there was a positive and significant correlation between age and ΔOT (*r* = 0.49, *t*(85) = 3.18, *p* = 0.001; Figure 2).

Replicating our previous finding (Barraza and Zak, 2009), changes in ACTH were positively correlated with higher scores on empathic concern after the video (*r* = 0.24, *t*(93) = 1.77, *p* = 0.04). Age was not correlated with changes in ACTH (*r* = −0.10, *t*(93) = 0.41, *p* = 0.34) or CORT (*r* = 0.09, *t*(80) = 0.35, *p* = 0.36). As expected, the changes in CORT and ACTH had a positive association (*r* = 0.47, *t*(80) = 3.21, *p* = 0.001). Basal ACTH trended toward a negative age gradient (*r* = −0.17, *t*(93) = 1.35, *p* = 0.09), but basal CORT did not (*r* = 0.05, *t*(80) = 0.10, *p* = 0.54).

#### Robustness Check

In order to confirm our OT results, we assayed OT in a random subsample of 36 participants for whom we had extra plasma using an RIA with extraction, as suggested in McCullough et al. (2013). The data revealed a trend for the positive correlation between ΔOT and age using the RIA (*r* = 0.24, *t*(34) = 1.05, *p* = 0.075, one-tailed). This test is underpowered due to the low sample size but provides additional evidence for the main effect. Matching the finding above, there was no overall change in ΔOT due to the video using the RIA (*M* = 0.25%, SD = 0.86, *t*(34) = 0.24, *p* = 0.81, *N* = 35).

### Donations to Charity

**FIGURE 3:**
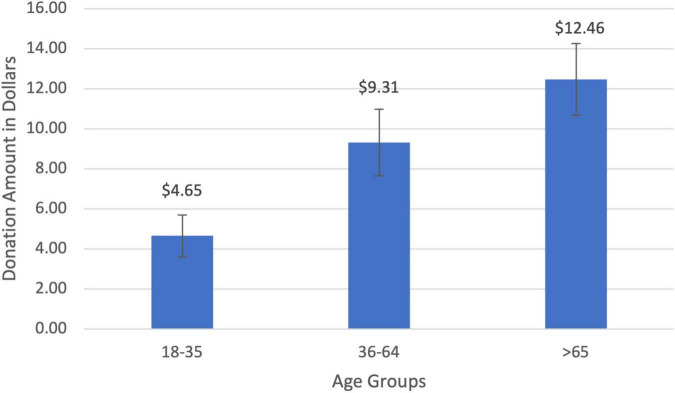
Charitable donation amount by age groups. There was a significant positive correlation between donation amount and age (*r* = 0.32, *p* = 0.001).

The average amount donated to charity was $8.19 (SD = $0.89). Charitable donations for the young age group (18–35) were $4.64 (SD = $1.05), increasing to $9.30 (SD = $1.66) for middle-aged participants (36–64), and rising to $12.45 (SD = $1.79) for seniors (>65; Figure 3). There was a significant positive relationship between age and the amount donated (*r* = 0.32, *t*(85) = 3.18, *p* = 0.001). Middle-aged participants donated twice as much to charity compared to young participants ($4.65; *t*(25) = 1.58, *p* = 0.06) while seniors donated 168% more than the young cohort ($7.80; *t*(23) = 3.26, *p* = 0.001). The age gradient for charitable giving was corroborated by two other measures of prosocial behaviors that increased with age: the amount of time one volunteered to help others (*r* = 0.19, *t*(89) = 1.57, *p* = 0.06) and the percentage of income donated to charity (*r* = 0.29, *t*(92) = 2.36, *p* = 0.01).

Consistent with the previous literature (Barraza and Zak, 2009), participants who had increases in both OT and ACTH (*N* = 40) donated 68% more to charity than participants (*N* = 63) who did not have these responses (OT^+^ & ACTH^+^: *M* = $12.10; Others: *M* = $7.19, *t* = 2.62, *p* = 0.01). Correlations for the amount donated to charity with the change in ACTH, OT, and CORT alone were not significant (*ps* > 0.05).

**FIGURE 4:**
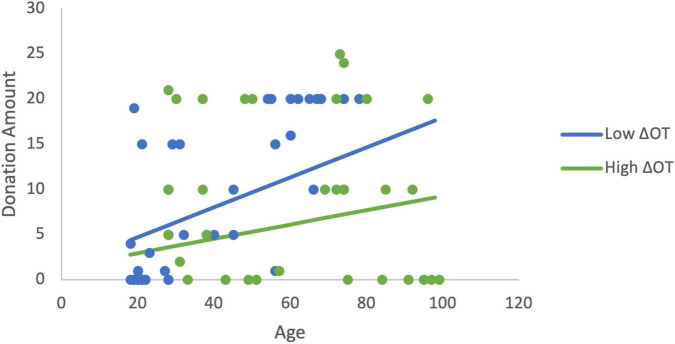
The moderation estimation shows that age has a larger effect on donations to charity when ΔOT is low while the effect of age on donations is positive but has a flatter gradient when ΔOT is high. The low value of ΔOT is the 40th percentile (4.05) and the high value is the 75th percentile (160.96).

In order to more fully examine the relationship between donations, age, and the changes in ACTH and OT, a set of least squares regression models were estimated. Estimating the linear effects of the changes in neurochemicals and age, only age was significantly related to donations. We hypothesized based on the findings above that age would moderate the effects of ΔOT. The initial estimation identified a mis-specification when both ΔOT and ΔOT*Age were included as explanatory variables due to multicollinearity (ΔOT variable inflation factor VIF > 5). As a result, ΔOT was dropped from the estimation in order to focus on the moderator. Note that this changes the interpretation of the interaction term (Faraway, 2004). In this estimation, the moderator (β = −0.0006, *p* = 0.029), and age are statistically significant and the model explains 20% of the variance in donations (*R*^2^ = 0.20, *F*(3,60) = 5.03, *p* = 0.004). The age gradient for donations is steeper when ΔOT is low compared to the slope when ΔOT is high (Figure 4). The variables continue to be significant (*R*^2^ = 0.21, *F*(5,58) = 3.09, *p* = 0.015) when sex and RCI are included as controls (moderator: β = −0.0005, *p* = 0.05).

#### Robustness Tests: Additional Prosocial Behaviors

Three additional prosocial behaviors were examined to assess the robustness of the charitable donation finding. These are donations to charity of money, goods, and time over the past year. Ordered logits were estimated for the three dependent variables using the same independent variables as in the immediate donation equation. As in the immediate donation equation, ΔOT was excluded due to multicollinearity (VIF > 6) with the moderator ΔOT*Age.

The past year’s donation of money to charity showed a positive age gradient (*r* = 0.38, *p* < 0.001). Estimating an ordered logit moderation model with ΔACTH, age, and ΔOT*Age, we found that OT*Age (β = −0.0001, *p* = 0.024) was a significant predictor in the model (χ^2^ = 19.71, *p* = 0.000). The positive age gradient holds for both high and low ΔOT with a steeper gradient when ΔOT is low rather than high (Appendix Figure A2). This relationship continued to be significant when including RCI and sex (OT*Age: χ^2^ = 24.15, *p* = 0.000).

The next measure of prosociality tested was goods donated to charity during the last year. As with money donated to charity, there was positive age gradient (*r* = 0.36, *p* < 0.001) for donated goods. Estimating the same ordered logit moderation model, OT*Age (β = −0.0001, *p* = 0.051) was a significant predictor in the model (*χ*^2^ = 16.62, *p* = 0.001). This relationship remained significant when accounting for RCI and sex (OT*Age: χ^2^ = 20.14, *p* = 0.001) and increased with age for both high and low ΔOT with a steeper gradient when ΔOT was low (Appendix Figure A3).

The third measure of prosociality was the number of times a participant volunteered at a social sector organization. Older participants trended toward having volunteered more often over the last year compared to younger people in the sample (*r* = 0.19, *p* = 0.065). The logit moderation model showed that that OT*Age (β = −0.0002, *p* = 0.002) was associated with volunteering (χ^2^ = 15.56, *p* = 0.001) and the relationship was robust to the inclusion of RCI and sex (OT*Age: χ^2^ = 21.13, *p* = 0.001). The positive relationship between age and volunteering is sustained for both low and high ΔOT matching the other two retrospective prosocial measures (Appendix Figure A4).

### Life Satisfaction and Religiosity

**FIGURE 5:**
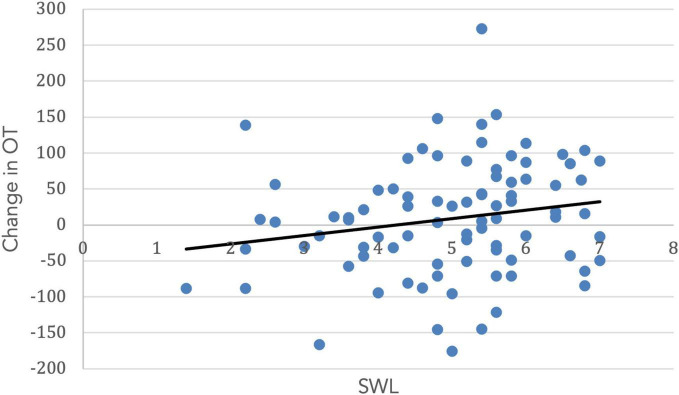
The change in OT had a positive correlation with satisfaction with life (*r* = 0.20, *p* = 0.04).

**FIGURE 6:**
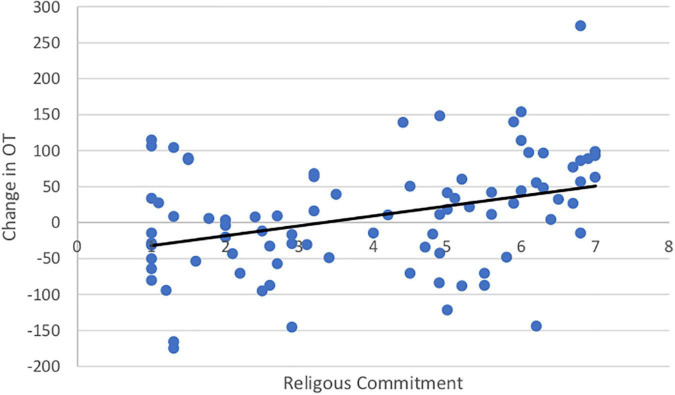
Positive correlation between religious commitment and change in OT.

Both SWL (*r* = 0.19, *t*(99) = 1.67. *p* = 0.05) and RCI (*r* = 0.54, *t*(99) = 3.11, *p* = 0.001) increased with age. Importantly, the change in OT was positively associated with SWL (*r* = 0.20, *t*(85) = 1.42, *p* = 0.04; Figure 5) and religious commitment (*r* = 0.41, *t*(85) = 3.17, *p* = 0.001, Figure 6). The SWL finding stands out because the frequency of contact with friends and loved ones fell with age (*r* = −0.28, *t*(92) = 2.36, *p* = 0.01). ΔOT was also associated with two dispositional traits, gratitude (*r* = 0.23, *t*(85) = 2.10, *p* = 0.019) and empathic concern (IRI, *r* = 0.28, *t*(79) = 1.90, *p* = 0.015).

## Discussion

Our analysis has identified a likely neurochemical impetus for prosocial behavior that remains intact with age. The data showed that older participants experienced the largest change in OT in response to an emotional stimulus compared to other age groups. The correlation between neurochemical changes and four measures of prosocial actions suggest that OT impacts prosocial behaviors more strongly in aging adults for small increases in OT. As in previous research, our data show that individuals who dispositionally have high empathic concern have a larger increase in OT after a video prime with social content (Barraza and Zak, 2013; Zak and Barraza, 2013). This dispositional effect partially dampens the age effect on OT from the prime revealing a trait-state interaction that influences the acute donation decision. The “high oxytocin responder” effect has been found for other stimuli and behaviors (Rameson et al., 2012; Procyshyn et al., 2020) and has been previously reported for the video used here (Barraza and Zak, 2009). Nevertheless, the positive age gradient for age on donations was maintained for both low and high ΔOT responders. Note that while there was no average change in OT for the video as in a previous study using the same stimulus (Barraza and Zak, 2009), in most published research using social stimuli to induce OT release, including studies with very large sample sizes, only about 50% of participants will show an increase (Barraza and Zak, 2013; Terris et al., 2018).

The other three retrospective measures of prosocial behaviors also had a positive moderating effect with age and show the same pattern vis-a-vis the magnitude of ΔOT. The retrospective measures of prosocial behaviors were included to increase the likelihood of inferential validity of the statistical test of the observed prosocial donation. That all three measures confirm the relationship with ΔOT is remarkable since retrospective measures are subject to more bias than directly observable behaviors. We believe the relationship between an acute change in OT and retrospective prosocial behaviors is new to the literature and should be replicated to ensure it does not only occur for the sample we collected. This also supports the positive correlation of ΔOT with the self-reports for life satisfaction, trait gratitude, and religious commitment. The larger prosocial response for the ΔOT*Age moderator when ΔOT is low demonstrates the powerful impact of age on helping behaviors.

An additional contribution of the present research is the demonstration that our results hold when analyzing the change in OT using unextracted OT from an ELISA as well as an extracted sample using an RIA. The extracted RIA change in OT trended toward a positive correlation with age (*r* = 0.27, *p* = 0.075). The post-stimulus OT values from the RIA and ELISA were positively correlated (*r* = 0.08, paired *t*(25), *p* = 0.0001) as in animal research (Valstad et al., 2017), though the change in OT across assays was not correlated (*r* = 0.05, paired *t*(25), *p* = 0.39).

Age moderated the relationship between ΔOT and immediate donations as well as the history of donations of money and goods. These associations continued to be significant when accounting for biological sex and religious participation. Our findings suggest a possible neurologic explanation for why older adults donate a higher portion of their income to charity (Simmons and Emanuele, 2004; Omoto et al., 2009; Choi and Chou, 2010; Bekkers and Wiepking, 2011). Participants who had positive changes in both ACTH and OT donated 68% more to charity than did those without these responses. The ACTH response is the peripheral signature of attention while the OT response indicates a participant’s emotional resonance with the narrative (Born et al., 1986; Barraza and Zak, 2013). This finding replicates our previous work on immediate donations (Barraza and Zak, 2009; Lin et al., 2013) and extends this to one’s history of donations. Our finding is surprising as older adults tend to be more distracted than younger people (Guerreiro et al., 2010), including while watching videos (Campbell et al., 2015), an area that requires more research. The larger OT response to the video among older adults may be due to heighted subjective emotional responses relative to young adults when viewing films about loss (Kunzmann and Grühn, 2005).

The moderated interaction of OT and age extended to volunteering as well. Seniors have been shown to volunteer more often than younger people (Castle et al., 2012; Sze et al., 2012; Wiles and Jayasinha, 2013; Beadle et al., 2015; Matsumoto et al., 2016). The parametric relationship for volunteering along with the other prosocial behaviors, reveals the strength of the relationship of OT and age. OT administration has been shown to increase dispositional gratitude (Barraza et al., 2013) and the findings here extend this relationship to the release of endogenous OT. Those with higher dispositional gratitude tend to engage in more helping behaviors than those who are less grateful (Bartlett and DeSteno, 2006). OT also functions as an analgesic (González-Hernández et al., 2014). Seniors who suffer from pain, which often reduces physical activity, tend to have fewer social interactions leading to isolation and increased risk of depression (Williamson and Schulz, 1992). Since positive social interactions are an OT stimulus (Barraza and Zak, 2013), there is likely a bidirectional effect of social activities and prosocial behaviors.

The heightened OT response by seniors was associated with an enhanced evaluation of life satisfaction compared to younger participants. While SWL has been shown to increase after middle age (Diener, 2009; Hansen and Slagsvold, 2012; Jivraj et al., 2014), we found that the variation in OT release was linearly associated with SWL. Religiosity is among the factors known to increase SWL (Bergan and McConatha, 2001; Diener and Clifton, 2002) and our analysis found a positive correlation between religious commitment (RCI) and the change in OT. The joint relationship of OT release on SWL, gratitude, and religiosity can help explain why seniors enjoy greater affective well-being than do younger adults (Scheibe and Carstensen, 2010). An innovation in our work is showing the need for a social stimulus to induce OT release so that seniors gain the psychological benefits we identified. Older adults often suffer from loneliness (Cacioppo and Patrick, 2008) that can reduce the social opportunities to release OT.

There are several limitations of the present study. We are unable to establish the causal relationship between OT, prosocial behaviors, and dispositional reports even while our analysis supports the hypotheses that OT influences prosocial behaviors and SWL in older adults. There are likely factors in addition to the release of OT that cause people to share money, donate to charity, participate in religious activities, and have high SWL that we were unable to measure and should be explored in future research. Further, prosocial behaviors may influence the likelihood and amount of OT release (Choi and Kim, 2011; Zak, 2012; Boenigk and Mayr, 2016). Findings in animals suggest a bi-directional causality in which OT release motivates prosocial behaviors that potentiate neural networks that influence future OT excretion (Nelson and Panksepp, 1998). As in nearly all published studies of endogenous OT release, the video stimulus was only effective for some participants that can be due to inattention, stress or interactions with other neurochemicals that additional measures could uncover (Zak et al., 2005; Neumann and Landgraf, 2012; Zak, 2012; Terris et al., 2018). The findings here should be considered preliminary since the sample size is small compared to other published research relating age to prosocial behaviors (Foulkes et al., 2018; Mayr and Freund, 2020). The sample is also geographically homogeneous which might have biased the results even while the main findings continue to hold with the inclusion of covariates such as religious practice. While measuring changes in oxytocin via serial blood draws is the gold standard, this makes large samples logistically difficult. An approach future research could use would be to capture the effect of oxytocin non-invasively for a larger sample through oxytocin’s activation of the vagus nerve using an electrocardiogram (Norman et al., 2011; Barraza et al., 2015). This will allow researchers to extend the sample size to confirm the results reported here.

The confluence of our findings relating OT to prosocial behaviors and the age gradient we report, are a bulwark against expectations that seniors are socially disengaged. Family members, clinical teams, activity directors, and seniors themselves should seek out more opportunities for social activities, even when mobility or pain limits activity choices. Seniors retain the ability and interest in social activity and its positive impact on health and happiness are well-established (Koopmans et al., 2010).

## Data Availability Statement

The datasets presented in this study can be found in online repositories. The names of the repository/repositories and accession number(s) can be found below: The data are available at Open IR openicpsr-147581.

## Ethics Statement

The studies involving human participants were reviewed and approved by the Claremont Graduate University Institutional Review Board (IRB # 1255). The patients/participants provided their written informed consent to participate in this study.

## Author Contributions

PZ and JB conceived and designed the study and collected data. PZ, JB, TO, and BC performed the analysis, interpreted the results, and prepared the manuscript. PZ obtained funding. All authors reviewed the results and approved the final version of the manuscript.

## Conflict of Interest

BC was employed by TripActions. The remaining authors declare that the research was conducted in the absence of any commercial or financial relationships that could be construed as a potential conflict of interest.

## Publisher’s Note

All claims expressed in this article are solely those of the authors and do not necessarily represent those of their affiliated organizations, or those of the publisher, the editors and the reviewers. Any product that may be evaluated in this article, or claim that may be made by its manufacturer, is not guaranteed or endorsed by the publisher.

## Figures and Tables

**APPENDIX A1-A4:**
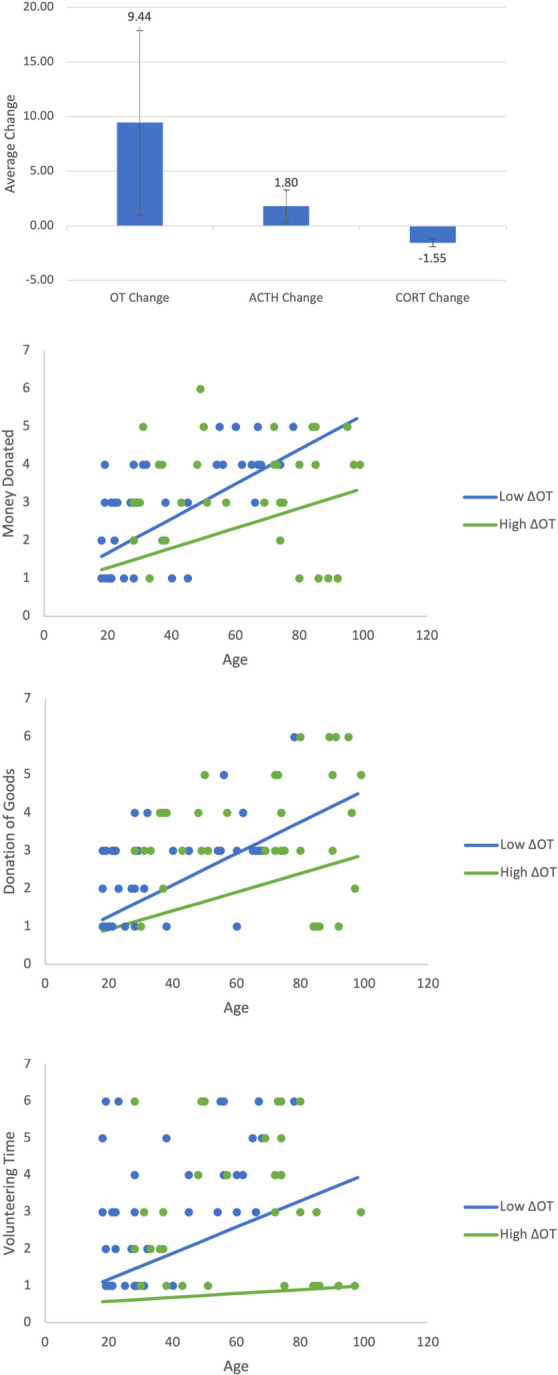
Shows the change in all three neurochemicals in response to the video stimulus. Show the moderating effect of age and ΔOT on retrospective prosocial behaviors. All three figures show that age increases prosocial behaviors irrespective of whether the positive change in ΔOT is high or low. This pattern is similar to that found for immediate donations. As in Figure 4, the low value of ΔOT is the value at the 40th percentile (4.05) and the high value is the 75th percentile (160.96).
